# Surgical treatment for cognitive impairment caused by internal jugular vein stenosis: a clinical study of atlas transverse process resection

**DOI:** 10.3389/fneur.2026.1776658

**Published:** 2026-03-11

**Authors:** Xupeng Peng, Junpeng Xu, Shuaibin Lu, Haiyang Ma, Sheng Xu, Meng Lv, Guangtong Zhu, Yuchuan Ding, Xunming Ji, Zhiqiang Hu

**Affiliations:** 1 Department of Neurosurgery, Beijing Shijitan Hospital, Capital Medical University, Beijing, China; 2 Department of Neurosurgery, Wayne State University School of Medicine, Detroit, MI, United States; 3 Department of Neurology and China-America Institute of Neuroscience, Xiongan Xuanwu Hospital, Baoding, China; 4 Department of Neurology, Xuanwu Hospital Capital Medical University, Beijing, China; 5 Beijing Advanced Innovation Center for Big Data-Based Precision Medicine, Beihang University, Beijing, China

**Keywords:** atlas transverse process, cognitive dysfunction, internal jugular vein stenosis, montreal cognitive, surgical decompression

## Abstract

**Background and objectives:**

Internal jugular vein (IJV) stenosis, often caused by extrinsic compression from the atlas transverse process, may impair cerebral venous drainage and contribute to cognitive dysfunction, though direct clinical evidence is limited. This study aimed to investigate the correlation between atlas-induced IJV stenosis and cognitive impairment, and to evaluate the effect of surgical decompression on cognitive outcomes.

**Methods:**

From January to June 2025, 47 patients with radiologically confirmed IJV stenosis due to atlas transverse process compression were prospectively enrolled. Cognitive function was assessed using the Montreal Cognitive Assessment (MoCA). Patients were stratified into higher (*n* = 24) and lower (*n* = 23) stenosis groups based on the mean of IJV stenosis degree (IJVS degree) for exploratory comparison. Of these, 16 patients completed the strict postoperative outpatient follow-up, including MoCA assessment.

**Results:**

The mean MoCA score for the cohort was 23.21 ± 3.41, indicating cognitive impairment. The higher stenosis group had significantly lower MoCA total scores than the lower group (20.79 ± 0.56 vs. 25.74 ± 0.38, *p* < 0.001), with pronounced deficits in visuospatial/executive function and delayed recall. Partial correlation analysis, controlling for age and education level, revealed a significant negative correlation between IJVS degree and MoCA score (*r* = −0.66, *p* < 0.001). Postoperatively, the mean MoCA score improved from 22.56 ± 0.82 to 24.68 ± 0.64 (*p* < 0.001), with significant gains in visuospatial/executive function, attention, and delayed recall.

**Conclusion:**

This study indicates that internal jugular vein stenosis caused by atlas transverse process compression is a potentially treatable contributor to cognitive dysfunction. Surgical decompression improved cognitive function, providing direct evidence for the role of the cerebral venous system in cognitive health. Evaluation of patients with cognitive complaints should consider assessment of the head and neck venous system.

## 1 Introduction

The internal jugular vein (IJV) serves as a major conduit for cerebral venous drainage, playing a vital role in the removal of metabolic waste, the reabsorption of cerebrospinal fluid, and the regulation of intracranial pressure (1). Stenosis of the IJV increases cerebral venous outflow resistance, thereby impairing venous drainage function (2).

A growing body of evidence suggests an association between cognitive decline and impaired cerebral venous drainage. For instance, jugular venous reflux, which is believed to compromise venous outflow and elevate cerebral venous pressure, has been correlated with cognitive deficits in several studies (3, 4). Similarly, heart failure leads to systemic venous hypertension and increased resistance to cerebral venous drainage, with cognitive impairment being a recognized complication (5–7). Animal models also support this link, as mice subjected to jugular vein ligation exhibit significant cognitive deterioration (8, 9). Furthermore, Pardo et al. (10) reported that patients with Alzheimer’s disease and mild cognitive impairment have a notably smaller cross-sectional area of the cerebral venous drainage system compared to healthy controls. Collectively, these findings indirectly suggest that compression and stenosis of the IJV — the primary drainage pathway — may substantially affect cognitive function. However, a direct clinical correlation between IJV stenosis and cognitive impairment has not been firmly established, with current literature limited mostly to isolated case reports (11).

IJV stenosis can result from intravascular or extrinsic causes. Extrinsic compression, often due to adjacent anatomical structures such as bone, arteries, or soft tissues, is a common mechanism. Among these, osseous compression — particularly from the atlas transverse process — is the most prevalent (12, 13). Severe compression by the atlas transverse process can even lead to apparent flow interruption on imaging, underscoring its clinical significance. This form of stenosis provides a unique, naturally occurring human model of isolated impairment in cerebral venous drainage. It enables the examination of the relationship between IJV stenosis and cognitive function without the confounding influence of conditions such as heart failure or venous reflux. Moreover, such stenosis can be alleviated surgically via resection of the atlas transverse process (14), allowing for the evaluation of cognitive changes before and after intervention, and offering a bidirectional perspective on the potential link between IJV patency and cognitive performance.

Therefore, this study aimed to directly assess cognitive function in patients with atlas-induced IJV stenosis, to investigate the impact of such stenosis on cognition, and to explore whether surgical decompression via atlas transverse process resection leads to cognitive improvement—thereby strengthening the evidence chain linking IJV stenosis to cognitive impairment.

## 2 Methods

### 2.1 Study population

Patients were prospectively enrolled from the Department of Neurosurgery at Beijing Shijitan Hospital between January 2025 and June 2025. Inclusion criteria were: (1) age between 18 and 75 years; (2) computed tomography venography (CTV) confirmation of internal jugular vein (IJV) stenosis caused by compression from the atlas transverse process; (3) no prior history of cerebral infarction, intracranial hemorrhage, Alzheimer’s disease, or other disorders known to cause cognitive decline; (4) availability of complete baseline clinical and imaging data; and (5) provision of written informed consent.

Exclusion criteria were: (1) presence of other venous abnormalities on imaging, such as venous sinus stenosis or jugular venous reflux, or patients in whom imaging identified the styloid process as the primary compressive structure causing IJV stenosis; (2) significant heart failure or other major cardiac dysfunction; (3) severe hearing impairment or other conditions that could compromise the validity or completion of cognitive assessments. (4) the identification of intrathoracic or intracranial space-occupying lesions, cerebrovascular malformations, or other abnormalities on high-resolution chest CT and brain MRI and CT that could constitute a major hemodynamic obstruction.

All enrolled patients underwent surgical decompression via atlas transverse process resection during their hospitalization.

### 2.2 Data and image collection

**Figure 1:**
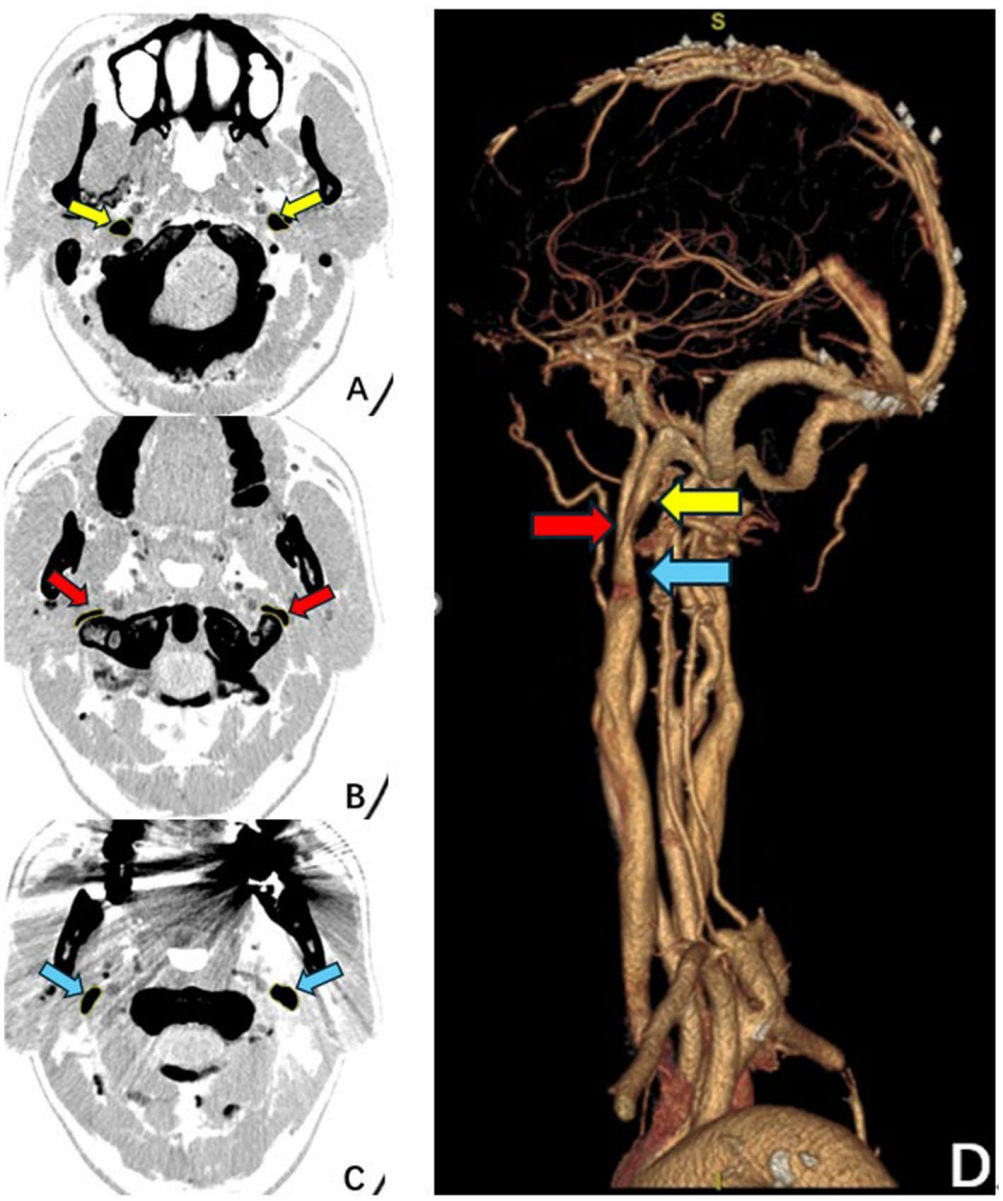
Schematic illustration of the measurement methodology for internal jugular vein stenosis. **(A–C)** Pre-operative axial computed tomography venography slices showing the three measurement planes used to calculate the relative stenosis degree (IJVS degree). **(D)** The three-dimensional reconstruction demonstrates the anatomical relationship, with arrows indicating the measurement sites. Arrow indicators: The red arrow corresponds to the cross-section at the narrowest segment **(B)**; the yellow arrow corresponds to the cross-section at the nearest proximal normal-appearing segment **(A)**; the blue arrow corresponds to the cross-section at the nearest distal normal-appearing segment **(C)**. The IJVS degree was calculated based on the average area of the proximal and distal normal segments relative to the area at the narrowest point.

Clinical characteristics and laboratory data were extracted from the hospital’s electronic medical record system, including: (1) demographic and clinical data (age, sex, years of education); and (2) imaging data. Imaging analysis was performed by a radiologist blinded to the study objectives. On CTV images, the cross-sectional areas of the IJV at specified bilateral levels were measured using Fiji software (ImageJ), as illustrated in Figure 1. The degree of IJV stenosis was quantified using a relative stenosis degree (IJVS degree), calculated as follows:


$$\text{IJVS degree}=[1-(\frac{S1+S2}{S3+S4+S5+S6})]\times 100\%$$

Where:

- S1: Residual area at the right-sided narrowest segment.
- S2: Residual area at the left-sided narrowest segment.
- S3: Normal area above the right-sided stenosis.
- S4: Normal area below the right-sided stenosis.
- S5: Normal area above the left-sided stenosis.
- S6: Normal area below the left-sided stenosis.

### 2.3 Cognitive function assessment and follow-up

Cognitive function was assessed by a neurologist who was blinded to the study objectives, using the Beijing version of the Montreal Cognitive Assessment (MoCA) scale. Assessments were conducted in a quiet room. All patients underwent a baseline evaluation preoperatively. A strict outpatient follow-up was performed 3 months after surgery, during which the MoCA assessment was repeated.

### 2.4 Surgical procedure

The decision to proceed with surgery in this study was based on systematic preoperative imaging evaluation. All patients underwent high-resolution CTV, which clearly identified the atlas transverse process as the primary compressive structure responsible for internal jugular vein stenosis. Consequently, all patients underwent targeted resection of the atlas transverse process and decompression of the J3 segment of the internal jugular vein (IJV). The surgery employed a standardized posterolateral microsurgical approach, the core principles and specific technical details of which have been previously elaborated in our literature (14). Briefly, the procedure involved the following key steps:

(1) Localization and exposure: The patient was placed in the lateral decubitus position. A small retroauricular/submastoid incision was made. Dissection proceeded along the anterior border of the sternocleidomastoid muscle to directly expose the C1 transverse process.
(2) Precise bony decompression: Under continuous irrigation and intraoperative neurophysiological monitoring, a high-speed drill was used to meticulously remove the compressive bony portion of the transverse process until restoration of pulsation and a lax appearance of the venous wall was achieved.
(3) Venous sheath release: Further anterior dissection involved opening the carotid sheath to release the soft tissues surrounding the IJV, ensuring complete decompression of the J3 segment.
(4) Cranial nerve protection: A dual strategy of anatomical avoidance (the posterolateral approach naturally avoids the anteromedial neurovascular bundle) and continuous intraoperative neurophysiological monitoring (focusing on muscles innervated by the accessory nerve) was employed to maximize cranial nerve safety.

**Figure 2:**
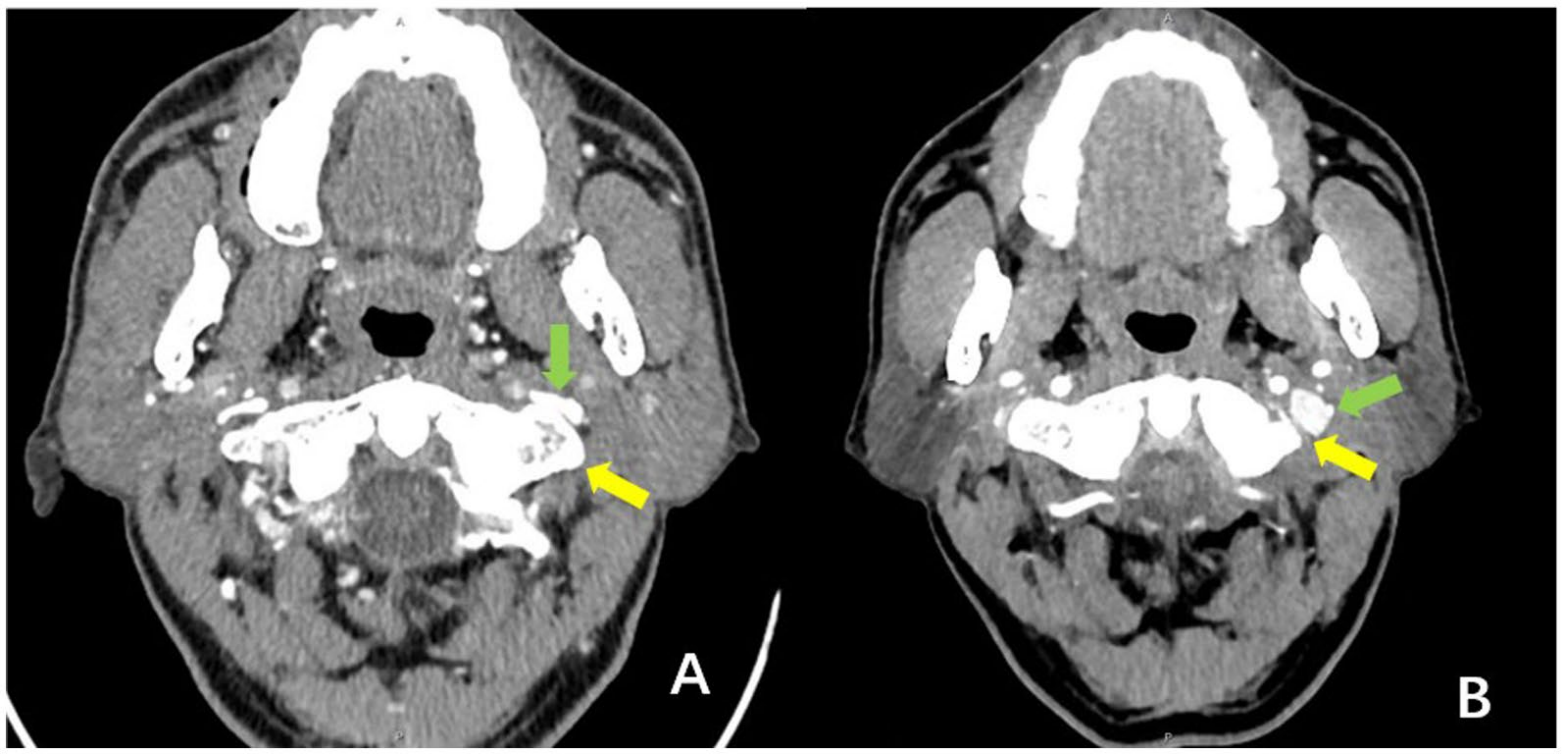
Comparative morphology of the internal jugular vein in a representative patient before surgery and 3 months postoperatively (axial CT venography views). **(A)** Preoperative image: Demonstrates protrusion of the atlas transverse process (yellow arrow), causing direct compression and significant luminal stenosis of the ipsilateral internal jugular vein (green arrow). **(B)** Postoperative (3-month) image: Shows resection of the compressive portion of the atlas transverse process (yellow arrow). The previously compressed segment of the internal jugular vein (green arrow) now exhibits substantial luminal expansion and restored morphology. Interpretation: This comparison provides direct visual evidence of successful surgical relief of the bony compression and subsequent anatomical restoration of the jugular vein.

Following decompression, restoration of IJV morphology was confirmed under direct microscopic and endoscopic visualization. This anatomical improvement was clearly demonstrated on CT venography at the 3-month postoperative follow-up, as shown in Figure 2.

### 2.5 Sample size estimation

Given the current lack of relevant research data in this field, this study estimated the required sample size based on the collected cohort data.

For the cross-sectional inter-group comparison, the sample size was estimated using the two independent samples *t*-test. The parameters were set as follows: the mean Montreal Cognitive Assessment (MoCA) score for the lower stenosis group was 20.79 ± 2.75 (mean ± standard deviation), and for the higher stenosis group, it was 25.74 ± 1.80. With a two-sided significance level of *α* = 0.05 and a statistical power of 90%, calculation using G*Power software indicated that only 5 patients per group were required to achieve the preset power. Considering an approximate 20% loss-to-follow-up rate, it was ultimately determined to recruit at least 6 patients per group, totaling a minimum of 12 patients.

To evaluate the effect of surgical intervention on cognitive improvement, the sample size was estimated using the paired-sample t-test. Parameters were based on a preoperative MoCA mean of 22.56 ± 3.28 and a postoperative mean of 24.68 ± 2.44, yielding a mean difference (Δ) of 2.12 points. The Pearson correlation coefficient between the pre- and postoperative scores was *r* = 0.813 (*p* < 0.001). Under the same significance level (*α* = 0.05, two-sided) and power (90%), calculation using G*Power software showed that nine complete pairs of data were needed. Similarly, accounting for a 20% loss-to-follow-up rate, it was determined that at least 11 patients needed to be recruited to undergo surgery and complete follow-up.

### 2.6 Statistical analysis

Statistical analyses were performed using SPSS software (version 27.0). Continuous data conforming to a normal distribution are presented as mean (SD), while non-normally distributed data are presented as median and interquartile range (IQR). Comparisons between two independent groups were conducted using the independent samples *t*-test or the Mann–Whitney U test, as appropriate. Comparisons between pre- and postoperative measurements within the same group were performed using the paired *t*-test or the Wilcoxon signed-rank test. A two-tailed *p* value < 0.05 was considered statistically significant.

### 2.7 Ethics statement

The authors affirm that all procedures contributing to this work complied with the ethical standards of the relevant institutional committee on human experimentation and with the Helsinki Declaration of 1975, as revised in 2013. The study protocol was reviewed and approved by the Local Ethics Committee of Beijing Shijitan Hospital, Capital Medical University (Approval No. IIT2025-021-003). All patients scheduled for surgical intervention were fully informed of the potential risks and benefits of the procedure. Written informed consent was obtained from all participants prior to their inclusion in the study.

## 3 Results

### 3.1 Baseline demographic and clinical characteristics

A total of 47 patients were enrolled in this study. The baseline characteristics of the entire cohort are summarized in Table 1. The cohort consisted of 26 males and 21 females, with a mean age of 52.66 years (SD = 11.98). The median duration of education was 12.00 years (IQR: 9.00–16.00). Common presenting symptoms include tinnitus (72%), subjective memory decline (62%), sleep disturbances (53%), and dry eyes (49%), among others, as detailed in Table 1, with an average symptom duration of approximately 46.60 months. Cognitive assessment using the MoCA revealed a mean total score of 23.21 (SD = 3.41) for the cohort, which is below the commonly accepted normal cutoff score of 26, indicating the presence of significant cognitive impairment in patients with IJV stenosis.

**Table 1:** Baseline characteristics of the entire patient cohort (*n* = 47).

	Total
Cases	47
Age, mean (SD)	52.66 (11.98)
Gender (Male/Female)	26/21
Years of education, median (IQR)	12.00 (9.00–16.00)
Area of the narrowest part, mean (SD)	0.60 (0.24)
IJVS degree, mean (SD)	0.28 (0.17)
MoCA, mean (SD)	23.21 (3.41)
Visuospatial/Executive, median (IQR)	3.00 (2.00–4.00)
Naming, median (IQR)	3.00 (3.00–3.00)
Attention, median (IQR)	5.00 (5.00–6.00)
Language, median (IQR)	2.00 (1.00–2.00)
Abstraction, median (IQR)	1.00 (1.00–2.00)
Delayed recall, median (IQR)	3.00 (1.00–4.00)
Orientation, median (IQR)	6.00 (6.00–6.00)
Head noise	33 (72%)
Sleep disturbance	25 (53%)
Tinnitus	22 (46%)
Dry or puffy eyes	23 (48%)
Headache	16 (34%)
Dizziness	17 (36%)
Neck discomfort	18 (38%)
Anxiety or depression	20 (42%)

### 3.2 Correlation between internal jugular vein stenosis and cognitive function

**Figure 3:**
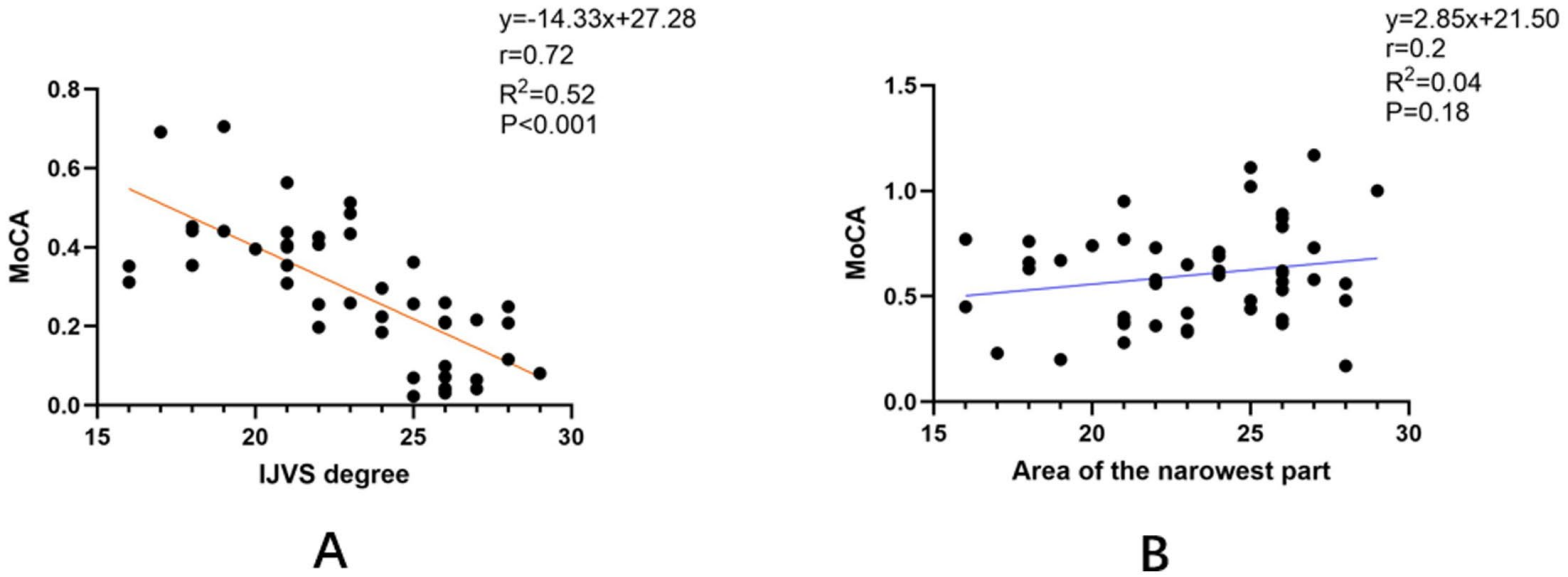
Scatter plots illustrating the correlations between cognitive function and two metrics of internal jugular vein stenosis in the total cohort (*n* = 47). **(A)** Correlation between the Internal Jugular Vein Stenosis degree (IJVS degree) and the Montreal Cognitive Assessment (MoCA) total score (*n* = 47). The solid line represents the linear regression fit. A significant negative Pearson correlation was observed (*r* = −0.72, *p* < 0.001). **(B)** Correlation between the cross-sectional area at the narrowest point (Area) and the MoCA total score (*n* = 47). No significant linear correlation was found (*r* = 0.20, *p* = 0.19).

Currently, there is no established guideline or grading standard for the severity of internal jugular vein (IJV) stenosis. We began by visually assessing the relationships between the MoCA total score and two key imaging metrics — the absolute cross-sectional area at the narrowest point and the internal jugular vein stenosis degree (IJVS degree)—using scatter plots (Figure 3). The scatter plots suggested a negative trend between the MoCA score and the IJVS degree, but no apparent visual association with the absolute cross-sectional area.

Since the normality test supported that the IJVS degree data followed a normal distribution (Shapiro–Wilk test, *p* = 0.057), we employed parametric tests for the subsequent analyses. To quantify these relationships, Pearson correlation analysis was performed. It confirmed a significant negative correlation between the MoCA total score and the IJVS degree (*r* = −0.72, *p* < 0.001), whereas no significant correlation was found with the absolute cross-sectional area at the narrowest point (*r* = 0.20, *p* = 0.19).

To further validate the discriminatory power of the IJVS degree, patients were divided into as higher stenosis group (*n* = 24) and a lower stenosis group (*n* = 23) based on its mean value. The two groups showed no statistically significant differences in baseline characteristics such as age, sex, and years of education (all *p* > 0.05, Table 2). However, the higher stenosis group had a significantly lower MoCA total score than the lower stenosis group (20.79 ± 2.75 vs. 25.74 ± 1.80, *p* < 0.001). Subgroup analysis revealed that the higher stenosis group also scored significantly lower in specific cognitive domains, including visuospatial/executive function (*p* = 0.002), and delayed recall (*p* < 0.001).

**Table 2:** Comparison between groups stratified by the mean IJVS degree.

	Lower stenosis groups	Higher stenosis groups	T/Z	*p*
Cases	23	24		
Age, mean (SD)	49.75 (12.31)	55.25 (11.05)	1.53	0.13
Gender,(Male/Female)	12/11	15/9	0.51	0.47
Years of education, median (IQR)	15.00 (12.00–16.00)	12.00 (9.00–15.00)	1.83	0.07
IJVS degree, median (IQR)	0.12 (0.06–0.22)	0.41 (0.35–0.45)	5.87	<0.001
MoCA, mean (SD)	25.74 (1.80)	20.79 (2.75)	7.22	<0.001
Visuospatial/Executive, median, (IQR)	4.00 (3.00–4.00)	2.00 (2.00–3.00)	3.11	0.002
Naming, median (IQR)	3.00 (3.00–3.00)	3.00 (3.00–3.00)	2.02	0.06
Attention median (IQR)	6.00 (5.00–6.00)	5.00 (5.00–6.00)	0.85	0.39
Language, median (IQR)	2.00 (2.00–2.00)	1.50 (1.00–2.75)	1.65	0.10
Abstraction, median (IQR)	1.00 (1.00–2.00)	1.00 (0.00–2.00)	0.18	0.85
Delayed recall, median (IQR)	4.00 (3.00–4.00)	1.00 (0.00–2.75)	4.75	<0.001
Orientation, median (IQR)	6.00 (6.00–6.00)	6.00 (6.00–6.00)	0.14	0.17

The plots demonstrate that cognitive performance is significantly associated with the relative degree of stenosis (IJVS degree) but not with the absolute cross-sectional area.

### 3.3 Correlation and partial correlation analyses

As shown in Table 3, univariate correlation analysis indicated that MoCA score was significantly correlated with age (*r* = −0.52, *p* < 0.001), years of education (*r* = 0.47, *p* < 0.001), and IJVS degree (*r* = −0.72, *p* < 0.001). To control for the confounding effects of age and education, a partial correlation analysis was performed. The result demonstrated that the IJVS degree remained significantly and negatively correlated with the MoCA score after adjustment (*r* = −0.66, *p* < 0.001) (Table 4).

**Table 3:** Univariate correlations between MoCA score and patient characteristics.

Variable	Correlation coefficient (*r*) with MoCA	*p*
Age	−0.52	<0.001
Gender	−0.57	0.70
Years of education	0.47	<0.001
IJVS degree	−0.72	<0.001

**Table 4:** Partial correlation between IJVS degree and MoCA score (controlling for age and years of education).

	MoCA	
IJVS degree	Correlation	−0.66
	Significance	<0.001

### 3.4 Changes in cognitive function from preoperative to 3-month postoperative assessment

All patients underwent atlas transverse process resection. At the 3-month postoperative follow-up, 16 patients completed reassessment. As shown in Table 5, the mean MoCA total score significantly improved from 22.56 preoperatively to 24.81 postoperatively (*p* < 0.001). Significant improvements were also observed specifically in the domains of Visuospatial/Executive functions (*p* = 0.007) and Delayed Recall (*p* = 0.02).

**Table 5:** Comparison of cognitive function before and 3 months after surgery (*n* = 16).

	Preoperative	3 months post-surgery	T/Z	*p*
Cases	16	16		
Age, mean (SD)	58.19 (14.45)			
Gender, (Male/Female)	11/5			
Years of Education, median (IQR)	12.00 (12.00–15.00)			
MoCA, mean (SD)	22.56 (3.28)	24.68 (2.44)	5.32	<0.001
Visuospatial/Executive, median, mean (SD)	3.00 (1.15)	3.68 (0.87)	3.15	0.007
Naming, median (IQR)	3.00 (3.00–3.00)	3.00 (3.00–3.00)	1.00	0.32
Attention, median (IQR)	5.50 (5.00–6.00)	5.50 (5.00–6.00)	1.00	0.32
Language, median (IQR)	2.00 (1.25–2.75)	2.00 (2.00–2.75)	1.73	0.083
Abstraction, median (IQR)	1.00 (1.00–2.00)	2.00 (1.00–2.00)	2.00	0.46
Delayed recall, mean (IQR)	2.50 (1.00–3.75)	3.00 (3.00–4.00)	2.23	0.02
Orientation, median (IQR)	6.00 (6.00–6.00)	6.00 (6.00–6.00)	1.00	0.32

### 3.5 Improvement of patient-reported symptoms at 3-month postoperative follow-up

Symptom status was systematically re-evaluated at the 3-month follow-up in the 16 patients. The preoperative prevalence and postoperative improvement rates for the major presenting symptoms are summarized in Table 6. When descriptively stratified by the preoperative stenosis severity (higher stenosis cohort, *n* = 7; lower stenosis cohort, *n* = 9), a trend suggesting a higher proportion of symptom improvement in the higher stenosis cohort was observed for several symptoms, including head noise, sleep disturbance, and headache. Given the limited sample size in each subgroup, formal statistical comparisons were not performed.

**Table 6:** Postoperative symptom improvement at 3-month follow-up (*n* = 16).

Symptom	Preoperative cases *n* (%)	Overall improvement *n* (%)*	Improvement in higher stenosis cohort (*n* = 7)^†^	Improvement in lower stenosis cohort (*n* = 9)^†^
Head noise	14 (87.5)	6 (42.9)	4/7 (57.1%)	2/7 (28.6%)
Sleep disturbance	14 (87.5)	5 (35.7)	4/7 (57.1%)	1/7 (14.3%)
Tinnitus/hearing loss	7 (43.8)	1 (14.3)	1/3 (33.3%)	0/4 (0.0%)
Ocular symptoms	12 (75.0)	2 (16.7)	2/6 (33.3%)	0/6 (0.0%)
Headache/heaviness	10 (62.5)	4 (40.0)	4/5 (80.0%)	1/5 (20.0%)
dizziness	9 (56.3)	6 (66.7)	4/4 (100.0%)	2/5 (40.0%)
Neck/shoulder discomfort	10 (62.5)	2 (20.0)	2/3 (66.7%)	0/7 (0.0%)
Anxiety/depression	13 (81.3)	4 (30.8)	4/6 (66.7%)	0/7 (0.0%)

^*^Overall Improvement Rate = (Patients with Improvement/Preoperative Cases) × 100%.

^†^Data are presented as: number of patients improved/number of patients with the symptom preoperatively within the subgroup (percentage). Formal statistical comparisons between cohorts were not performed due to the limited sample size in each subgroup.

## 4 Discussion

### 4.1 Impact of internal jugular vein stenosis on cognitive function

This study provides preliminary evidence indicating a negative correlation between the degree of internal jugular vein (IJV) stenosis, as quantified by our newly proposed metric (IJVS degree), and cognitive impairment, even after controlling for the effects of age and education (partial *r* = −0.66, *p* < 0.001). These findings lend support to the emerging concept that impaired craniocervical venous outflow may contribute to cognitive dysfunction. Furthermore, our observational data suggested a trend toward cognitive improvement following surgical decompression. These findings offer novel and direct evidence from the perspective of cerebral venous drainage, highlighting the involvement of venous system dysfunction in cognitive regulation and re-emphasizing the importance of the venous system.

Unlike previous studies that often focused on the absolute cross-sectional area of the IJV, this study introduces a methodological approach that addresses the limitation of absolute measurements by normalizing the stenosis degree to adjacent venous segments. This approach, conceptually similar to standardized methods for quantifying arterial stenosis (e.g., NASCET, WASID), suggests that cognitive impairment is more closely associated with this relative degree of stenosis than with the absolute cross-sectional area. This central finding is robustly supported by the strong negative correlation demonstrated in our scatter plot analysis (Figure 3) and the significant inter-group differences based on the same metric (Table 2). Specifically, a higher degree of stenosis (i.e., a greater percentage reduction in lumen area) correlates with more severe cognitive deficits, whereas the absolute lumen area alone is not a reliable predictor of cognitive changes. This finding aligns with prior conclusions suggesting that the absolute cross-sectional area at the jugular foramen level does not affect cognitive function (10). The anatomical basis for this lies in the rich collateral circulation and anatomical variability of the cerebral venous system (15, 16), which makes the absolute area at a single site an inadequate surrogate for the severity of functional impairment in venous outflow. Therefore, measuring the absolute cross-sectional area alone is insufficient to quantify the hemodynamic significance of a stenosis.

The study cohort exclusively comprised patients with extrinsic compression stenosis caused by the atlas transverse process. A characteristic of such lesions is the considerable difficulty in obtaining the “original normal area” at the exact level of the stenosis. Based on the anatomical principle that the internal jugular vein area increases continuously and gradually from the cranial to the caudal direction (17), and informed by established methodological approaches from prior research (12), we used the average cross-sectional area of the normal vessel segments immediately proximal and distal to the stenosis as a reasonable surrogate to estimate the “ideal original area” at that site. The relative stenosis degree calculated using this method proved to be a sensitive metric for assessing impaired venous drainage and its correlation with cognitive function.

This study not only establishes, for the first time at the clinical level, a quantitative relationship between IJV stenosis degree and cognitive function but, more importantly, offers a new methodological perspective for accurately assessing the hemodynamic impact of venous stenosis. It argues for prioritizing the evaluation of the relative stenosis rate over absolute measurements, which is particularly valuable for lesions like extrinsic compression where the original lumen cannot be determined.

### 4.2 Cognitive improvement following atlas transverse process resection and potential mechanisms

Our results show that patients’ cognitive function improved at the 3-month postoperative follow-up, with significant gains particularly in the domains of visuospatial/executive function and delayed recall. Notably, the pattern of cognitive change in the surgical group closely mirrored the differences observed between the higher and lower stenosis groups at baseline. This not only further corroborates that IJV stenosis is an objective factor affecting cognitive function but also suggests that impaired cerebral venous drainage may predominantly impact visuospatial/executive abilities and delayed memory.

The cognitive improvement likely stems from the restoration of cerebral venous drainage post-surgery. Reduced resistance in IJV outflow alleviates cerebral venous congestion (18, 19), thereby potentially improving cerebral perfusion and metabolism (20). Additionally, the restoration of blood–brain barrier integrity and the alleviation of neuroinflammation may also contribute to cognitive recovery (2).

The observed cognitive improvement following surgical decompression is mechanistically supported by potential restoration of cerebral venous outflow. Data from our team’s independent cohort (non-overlapping with the present study) indicate that atlas transverse process resection leads to substantial hemodynamic benefit: at 3 months postoperatively, 75.0% of patients achieved significant or moderate recovery in jugular venous flow, while only 25.0% showed no change. This high rate of flow improvement provides a plausible physiological substrate for the cognitive gains observed in our surgical cohort. Although comprehensive preoperative and postoperative ultrasonography was not a prespecified endpoint in the current study, we are prospectively collecting such hemodynamic data alongside cognitive assessments in an ongoing extended follow-up study to directly establish the correlation between venous flow restoration and cognitive outcome.

Furthermore, the cerebral venous system exhibits considerable redundancy. The internal jugular veins are not the sole outflow conduits, as the vertebral venous plexus serves as a significant alternative pathway, particularly in the upright position (21). Additionally, it is noteworthy that cerebral waste clearance involves not only venous drainage but also the recently characterized glymphatic system and meningeal lymphatic vessels (22, 23). While a detailed discussion of these lymphatic mechanisms is beyond the scope of this study, it is plausible that the improvement in jugular venous outflow post-surgery could secondarily facilitate interstitial fluid dynamics and glymphatic clearance, representing a promising avenue for future mechanistic research.

In summary, surgical intervention to improve IJV drainage in this study led to enhanced cognitive function, and the specific domains of improvement closely matched the impairment profile caused by the pre-existing stenosis. This further supports the notion that impaired cerebral venous drainage can cause cognitive deficits, potentially through mechanisms involving improved cerebral perfusion and metabolism, as well as modulation of neuroinflammation.

### 4.3 Other factors influencing cognitive function

Cognitive function, as a higher-order mental process that involves perceiving, interconnecting, and evaluating information about objective entities, is influenced by multiple factors. In addition to identifying internal jugular vein stenosis as a contributing factor to cognitive impairment, this study also confirmed the significant effects of age and educational attainment on cognitive performance. Specifically, cognitive scores tended to decrease with advancing age and increase with longer duration of formal education, which aligns with findings from prior research (24, 25). Consequently, enhancing educational opportunities may represent a viable public health strategy for improving cognitive reserve and function in the broader population.

### 4.4 Surgical safety, tolerability, and considerations for cervical stability

All patients tolerated the surgical procedure well, with no serious adverse events recorded. The only commonly reported postoperative symptom was mild and transient local swelling, which typically resolves spontaneously within 3 to 5 days after surgery. A specific and important safety consideration is the potential impact on cervical biomechanical stability following resection of the atlas transverse process. All procedures adhered to a principle of precise, limited decompression, removing only the laterally projecting portion of the bone directly compressing the IJV while preserving the lateral mass, transverse process base, and critical ligamentous attachments. Anatomically, the primary stabilizers of the atlantoaxial joint (C1–C2) are the transverse and alar ligaments, which have no direct mechanical connection to the resected lateral tip of the transverse process (26, 27). Consequently, this limited resection is theoretically expected to have minimal impact on craniocervical stability. This is supported by our short-term clinical follow-up, in which no new symptoms indicative of cervical instability (e.g., refractory neck pain, radiculopathy, or myelopathic signs) were observed. Therefore, within the context of this study and its follow-up period, atlas transverse process resection appears to be a safe intervention for eligible patients. Nonetheless, the long-term biomechanical safety, as assessed by dynamic imaging in future studies, warrants further investigation.

### 4.5 Future perspectives

This study provides preliminary evidence supporting a link between atlas-induced IJV stenosis and cognitive impairment, and suggests potential for surgical benefit. However, significant uncertainties remain within the logic chain, which should be the focus of future research. First, regarding the quantification of IJV stenosis, while the IJVS degree metric introduced here offers a reproducible, relative measurement method, its clinical utility and correlation with direct hemodynamic parameters require validation. Future studies should compare and standardize different imaging modalities (e.g., high-resolution ultrasonography, CTV, MR venography, and catheter venography) to establish a reliable and accessible diagnostic protocol. Second, to firmly establish the association between IJVS and cognitive dysfunction, larger-scale, multi-center prospective studies with more comprehensive control of confounding variables (e.g., cardiovascular risk factors, sleep quality, mood disorders) are needed. Employing more extensive neuropsychological test batteries beyond the MoCA could provide deeper insights into the specific cognitive domains affected. Third, the therapeutic effect of surgical decompression observed in our small cohort requires confirmation in larger, controlled trials with longer follow-up periods. Such studies should systematically incorporate pre- and postoperative hemodynamic assessments (e.g., quantitative flow imaging) to directly correlate anatomical decompression with physiological improvement and cognitive outcome. Finally, promoting the standardization of diagnostic criteria and the use of validated multidimensional symptom scales (e.g., the Cerebral Venous Disorders Severity Scale, CVDSS) will be crucial for refining patient selection and enabling more nuanced correlations between imaging parameters, symptom burden, and cognitive performance.

## 5 Limitations

This study has several limitations. First and foremost, the postoperative follow-up rate was suboptimal. Only 16 of the 47 operated patients (34%) completed the scheduled 3-month cognitive assessment. This high rate of loss to follow-up substantially limits the robustness and generalizability of our postoperative findings and introduces a potential for significant selection bias. The observed cognitive improvements must therefore be interpreted with caution, as the characteristics and outcomes of the non-followed patients remain unknown. Second, despite its prospective design, the final sample size—particularly within the surgical subgroup with complete follow-up—remains relatively limited. This may affect statistical power and constrain the exploration of more subtle effects or subgroup differences. Furthermore, the observed gender distribution (slight male predominance) in our surgical cohort differs from the female preponderance reported in some broader studies of IJV stenosis. This discrepancy may be influenced by our limited sample size, selection bias inherent to a single-center surgical series, or potentially distinct characteristics of the specific patient subgroup undergoing intervention for atlas-induced compression. Third, the interrater reliability of the IJVS degree measurement was not assessed. While measurements were performed by a single, blinded radiologist to ensure internal consistency, this design precluded an evaluation of measurement variability across different observers. Thus, the reproducibility of this metric remains to be established. Fourth, the CTV-based measurement of IJV cross-sectional area is susceptible to technical confounders. Variables such as patient hydration status, the phase of respiration during image acquisition, and precise head/neck positioning can significantly influence venous diameter. These factors were not systematically controlled or recorded in our study, representing a potential source of measurement error. Fifth, there is currently no universally accepted, objective gold standard for quantifying the degree of internal jugular vein stenosis. Although the relative stenosis ratio used in this study is anatomically reasoned, its measurement still involves a degree of subjectivity, which may affect the comparability of results across different centers. Sixth, the imaging evaluation in this study was primarily designed for surgical planning and exclusion of complications. Although postoperative images (e.g., Figure 2) visually suggest anatomical improvement of the internal jugular vein, we lack standardized, systematic quantitative analysis of metrics such as venous diameter, cross-sectional area, or flow velocity. This limitation prevents precise quantification of the degree of anatomical decompression achieved by surgery and hinders a deeper exploration of the dose–response relationship between anatomical improvement and clinical symptom relief. Seventh, the assessment of venous hemodynamics in this study relied primarily on anatomical imaging (CTV). Although adjunctive tests like TCD and MRI were employed, we did not systematically perform quantitative flow imaging (e.g., phase-contrast MRV) or invasive pressure measurements. Consequently, we lack direct postoperative physiological data within this cohort to confirm that the intended improvement in venous outflow was achieved. Furthermore, the postoperative follow-up period was relatively short (3 months), and the absence of a non-surgical control group makes it difficult to assess the long-term stability of cognitive improvements or to fully exclude potential placebo effects or natural fluctuations.

Nonetheless, this study is the first to systematically demonstrate, at the clinical level, a significant association between internal jugular vein stenosis caused by a specific anatomical factor (atlas transverse process compression) and cognitive dysfunction, and to preliminarily confirm that surgical decompression can lead to cognitive improvement. These findings provide novel, direct evidence for the pathological mechanism of “venous cognitive impairment.” Future studies should employ larger-scale, multicenter prospective designs, incorporate longer follow-up periods and control groups, and develop more objective methods for quantifying stenosis and assessing hemodynamics to further validate and extend the findings of this research.

## 6 Conclusion

This study indicates that internal jugular vein stenosis caused by atlas transverse process compression is a potentially treatable contributor to cognitive dysfunction. Surgical decompression improved cognitive function, providing direct evidence for the role of the cerebral venous system in cognitive health. Evaluation of patients with cognitive complaints should consider assessment of the head and neck venous system.

## Data Availability

The datasets presented in this article are not readily available because patient data comes from the hospital information center and must be approved by the researchers. Requests to access the datasets should be directed to XP, 19944602350@163.com.

## References

[1] Zivadinov R Chung CP. Potential involvement of the extracranial venous system in central nervous system disorders and aging. BMC Med. (2013) 11:260. doi: 10.1186/1741-7015-11-260, 24344742 PMC3866257

[2] Mayhan WG Heistad DD. Role of veins and cerebral venous pressure in disruption of the blood-brain barrier. Circ Res. (1986) 59:216–20. doi: 10.1161/01.RES.59.2.216, 3742745

[3] Chung CP Beggs C Wang PN Bergsland N Shepherd S Cheng CY . Jugular venous reflux and white matter abnormalities in Alzheimer's disease: a pilot study. J Alzheimers Dis. (2014) 39:601–9. doi: 10.3233/JAD-131112, 24217278

[4] Beggs C Chung CP Bergsland N Wang PN Shepherd S Cheng CY . Jugular venous reflux and brain parenchyma volumes in elderly patients with mild cognitive impairment and Alzheimer's disease. BMC Neurol. (2013) 13:157. doi: 10.1186/1471-2377-13-157, 24176095 PMC4228414

[5] Kong S Gootee E Williams N Gottesman RF Johansen MC. Congestive heart failure and its associations with cognition and cerebral blood flow. Brain circulation. (2025) 11:30–8. doi: 10.4103/bc.bc_86_24, 40224550 PMC11984822

[6] Jefferson AL Himali JJ Beiser AS Au R Massaro JM Seshadri S . Cardiac index is associated with brain aging: the Framingham heart study. Circulation. (2010) 122:690–7. doi: 10.1161/CIRCULATIONAHA.109.905091 20679552 PMC2929763

[7] Cornwell WK 3rd Levine BD. Patients with heart failure with reduced ejection fraction have exaggerated reductions in cerebral blood flow during upright posture. JACC Heart failure. (2015) 3:176–9. doi: 10.1016/j.jchf.2014.10.006, 25543968

[8] Fulop GA Ahire C Csipo T Tarantini S Kiss T Balasubramanian P . Cerebral venous congestion promotes blood-brain barrier disruption and neuroinflammation, impairing cognitive function in mice. GeroScience. (2019) 41:575–89. doi: 10.1007/s11357-019-00110-1, 31691147 PMC6885079

[9] Wei H Jiang H Zhou Y Xiao X Zhou C Ji X. Cerebral venous congestion alters brain metabolite profiles, impairing cognitive function. J Cereb Blood Flow Metab. (2023) 43:1857–72. doi: 10.1177/0271678X231182244, 37309740 PMC10676144

[10] Pardo K Khasminsky V Keret O Benninger F Goldberg I Shelef I . Alzheimer's disease patients have smaller venous drainage system compared to cognitively healthy controls. Alzheimers Dement. (2025) 21:e14551. doi: 10.1002/alz.14551, 39936326 PMC11851167

[11] Primiani CT Lawton M Hillis AE Hui FK. Pearls & oy-sters: cerebral venous congestion associated with cognitive decline treated by jugular release. Neurology. (2022) 99:577–80. doi: 10.1212/WNL.0000000000201037 35851254

[12] Wang Z Ding J Bai C Ding Y Ji X Meng R. Clinical classification and collateral circulation in chronic cerebrospinal venous insufficiency. Front Neurol. (2020) 11:913. doi: 10.3389/fneur.2020.00913, 33071925 PMC7538781

[13] Zhou D Ding JY Ya JY Pan LQ Yan F Yang Q . Understanding jugular venous outflow disturbance. CNS Neurosci Ther. (2018) 24:473–82. doi: 10.1111/cns.12859, 29687619 PMC6489808

[14] Lu S Peng W Ma H Peng X Zhao R Xu S . Effectiveness of atlas transverse process resection combined with release and perfusion techniques in treating clinical symptoms caused by internal jugular vein stenosis secondary to external compression. World Neurosurg. (2025) 202:124397. doi: 10.1016/j.wneu.2025.124397, 40850666

[15] Pelizzari L Laganà MM Jakimovski D Bergsland N Hagemeier J Baselli G . Neck vessel cross-sectional area measured with MRI: scan-rescan reproducibility for longitudinal evaluations. J Neuroimaging. (2018) 28:48–56. doi: 10.1111/jon.12488, 29205670 PMC5760334

[16] Freitas CAF Santos L Santos AN Amaral Neto ABD Brandão LG. Anatomical study of jugular foramen in the neck. Braz J Otorhinolaryngol. (2020) 86:44–8. doi: 10.1016/j.bjorl.2018.09.004, 30348503 PMC9422587

[17] Buch K Groller R Nadgir RN Fujita A Qureshi MM Sakai O. Variability in the cross-sectional area and narrowing of the internal jugular vein in patients without multiple sclerosis. AJR Am J Roentgenol. (2016) 206:1082–6. doi: 10.2214/AJR.15.14689, 26958902

[18] Zhou D Meng R Zhang X Guo L Li S Wu W . Intracranial hypertension induced by internal jugular vein stenosis can be resolved by stenting. Eur J Neurol. (2018) 25:365–e13. doi: 10.1111/ene.13512, 29114973

[19] Marcotti S Marchetti L Cecconi P Votta E Fiore GB Barberio A . An anatomy-based lumped parameter model of cerebrospinal venous circulation: can an extracranial anatomical change impact intracranial hemodynamics? BMC Neurol. (2015) 15:95. doi: 10.1186/s12883-015-0352-y, 26099795 PMC4476203

[20] Huang CJ Zhou X Yuan X Zhang W Li MX You MZ . Contribution of inflammation and hypoperfusion to white matter hyperintensities-related cognitive impairment. Front Neurol. (2021) 12:786840. doi: 10.3389/fneur.2021.786840 35058875 PMC8763977

[21] Epstein HM Linde HW Crampton AR Ciric IS Eckenhoff JE. The vertebral venous plexus as a major cerebral venous outflow tract. Anesthesiology. (1970) 32:332–40. doi: 10.1097/00000542-197004000-00007, 4985574

[22] Chachaj A Gąsiorowski K Szuba A Sieradzki A Leszek J. The lymphatic system in the brain clearance mechanisms - new therapeutic perspectives for Alzheimer's disease. Curr Neuropharmacol. (2023) 21:380–91. doi: 10.2174/1570159X20666220411091332, 35410605 PMC10190136

[23] Louveau A Smirnov I Keyes TJ Eccles JD Rouhani SJ Peske JD . Structural and functional features of central nervous system lymphatic vessels. Nature. (2015) 523:337–41. doi: 10.1038/nature14432, 26030524 PMC4506234

[24] Lövdén M Fratiglioni L Glymour MM Lindenberger U Tucker-Drob EM. Education and cognitive functioning across the life span. Psychol Sci Public Interest. (2020) 21:6–41. doi: 10.1177/1529100620920576, 32772803 PMC7425377

[25] Seblova D Berggren R Lövdén M. Education and age-related decline in cognitive performance: systematic review and meta-analysis of longitudinal cohort studies. Ageing Res Rev. (2020) 58:101005. doi: 10.1016/j.arr.2019.101005, 31881366

[26] Kim D Viswanathan VK Munakomi S Menger RP. C1 Fractures. Treasure Island (FL): StatPearls Publishing LLC (2025). 30475564

[27] Karaaslan B Börcek A Uçar M Aykol Ş. Can the etiopathogenesis of Chiari malformation be Craniocervical junction stabilization difference? Morphometric analysis of Craniocervical junction ligaments. World Neurosurg. (2019) 128:e1096–101. doi: 10.1016/j.wneu.2019.05.072, 31103770

