# Correction: Surgical treatment for cognitive impairment caused by internal jugular vein stenosis: a clinical study of atlas transverse process resection

**DOI:** 10.3389/fneur.2026.1839312

**Published:** 2026-04-17

**Authors:** Xupeng Peng, Junpeng Xu, Shuaibin Lu, Haiyang Ma, Sheng Xu, Meng Lv, Guangtong Zhu, Yuchuan Ding, Xunming Ji, Zhiqiang Hu

**Affiliations:** 1 Department of Neurosurgery, Beijing Shijitan Hospital, Capital Medical University, Beijing, China; 2 Department of Neurosurgery, Wayne State University School of Medicine, Detroit, MI, United States; 3 Department of Neurology and China-America Institute of Neuroscience, Xiongan Xuanwu Hospital, Baoding, China; 4 Department of Neurology, Xuanwu Hospital Capital Medical University, Beijing, China; 5 Beijing Advanced Innovation Center for Big Data-Based Precision Medicine, Beihang University, Beijing, China

**Keywords:** atlas transverse process, cognitive dysfunction, internal jugular vein stenosis, Montreal Cognitive, surgical decompression

In the published article, the author order was erroneously given as:

Xupeng Peng^1^, Junpeng Xu^1^, Shuaibin Lu^1^, Haiyang Ma^1^, Sheng Xu^1^, Meng Lv^1^, Zhiqiang Hu^1^, Yuchuan Ding^2, 3^, Xunming Ji^4, 5^ and Guangtong Zhu^1*^

The updated author list appears below:

Xupeng Peng^1†^, Junpeng Xu^1†^, Shuaibin Lu^1^, Haiyang Ma^1^, Sheng Xu^1^, Meng Lv^1^, Guangtong Zhu^1^, Yuchuan Ding^2, 3^, Xunming Ji^4, 5*^, and Zhiqiang Hu^1*^

Additional changes include first authorship and equal contribution, this is marked with a symbol and the change appears below:

^†^These authors have contributed equally to this work and share first authorship

The corresponding author “Guangtong Zhu” was also changed.

The updated corresponding authors appear below:

Zhiqiang Hu neuro7@163.com, Xunming Ji jixm@ccmu.edu.cn

The original version of this article has been updated.

